# Lateral parabrachial FoxP2 neurons regulate respiratory responses to hypercapnia

**DOI:** 10.21203/rs.3.rs-2865756/v1

**Published:** 2023-05-05

**Authors:** Satvinder Kaur, Lynch Nicole, Yaniv Sela, Janayna Lima, Renner Thomas, Sathyajit Bandaru, Clifford Saper

**Affiliations:** Beth Israel Deaconess Medical Center; Beth Israel Deaconess Medical Center; Beth Israel Deaconess Medical Center; Beth Israel Deaconess Medical Center; Beth Israel Deaconess Medical Center; Beth Israel Department of Neurology, Program in Neuroscience and Division of Sleep Medicine, Beth Israel Deaconess Medical Center and Harvard Medical School, Boston, Ma-02215; Beth Israel Deaconess Medical Center

## Abstract

Although CGRP neurons in the external lateral parabrachial nucleus (PBel^CGRP^ neurons) are critical for cortical arousal in response to hypercapnia, activating them has little effect on respiration. However, deletion of all Vglut2 expressing neurons in the PBel region suppresses both the respiratory and arousal response to high CO2. We identified a second population of non-CGRP neurons adjacent to the PBel^CGRP^ group in the central lateral, lateral crescent and Kölliker-Fuse parabrachial subnuclei that are also activated by CO2 and project to the motor and premotor neurons that innvervate respiratory sites in the medulla and spinal cord. We hypothesize that these neurons may in part mediate the respiratory response to CO2 and that they may express the transcription factor, Fork head Box protein 2 (FoxP2), which has recently been found in this region. To test this, we examined the role of the PB^FoxP2^ neurons in respiration and arousal response to CO2, and found that they show cFos expression in response to CO2 exposure as well as increased intracellular calcium activity during spontaneous sleep-wake and exposure to CO2. We also found that optogenetically photo-activating PB^FoxP2^ neurons increases respiration and that photo-inhibition using archaerhodopsin T (ArchT) reduced the respiratory response to CO2 stimulation without preventing awakening. Our results indicate that PB^FoxP2^ neurons play an important role in the respiratory response to CO2 exposure during NREM sleep, and indicate that other pathways that also contribute to the response cannot compensate for the loss of the PB^FoxP2^ neurons. Our findings suggest that augmentation of the PB^FoxP2^ response to CO2 in patients with sleep apnea in combination with inhibition of the PBel^CGRP^ neurons may avoid hypoventilation and minimize EEG arousals.

## Introduction

Patients with obstructive sleep apnea (OSA) have recurring arousals over the course of a night with loss of the upper airway muscle tone that results in obstruction of the airway, causing a reduction (hypopnea) or cessation (apnea) of ventilation, despite persisting respiratory efforts. The interruption of ventilation causes progressive hypercapnia and hypoxia, which in turn causes increased ventilatory effort. This increased ventilatory effort is measured by increased activity (as measured by EMG) of muscles related to ventilation, including both airway dilators such as the genioglossus (GG) and pump muscles such as the diaphragm,^1–6^ and is associated ultimately with cortical arousal that further augments respiratory efforts that open up the airway and re-establish ventilation^5,7–9^. However, these repeated awakenings cause sleep fragmentation, which is associated with deleterious cognitive, metabolic and cardiovascular consequences^5,7,8,10–15^. The CO2 blood levels and ventilation increase rapidly during an apnea, but the fall in blood oxygenation typically lags behind the changes in CO2 blood levels and correlates poorly with the onset of the arousal^16–18^. We have therefore developed a mouse model of the repeated periods of exposure to elevated CO2 and brief arousal that mimics the events in OSA^19–21^. This model permits selective genetic targeting and manipulation of brain circuits that mediate the respiratory and EEG responses during the period of CO2 elevation associated with an apnea.

Our earlier work supports the hypothesis that the brain circuits that respond to hypercapnia by elevating respiratory efforts may be distinct from those that cause cortical arousal^19,20,22^. Specifically, we found that glutamatergic neurons (i.e., expressing Vglut2) in the lateral PB complex show cFos activation during elevated CO2, and that deletion of Vglut2 from these neurons in the lateral PB (LPB) caused both a delay in arousal and reduced ventilation to a CO2 stimulus^20^. However, the two aspects of CO2 responses were not correlated across our deletions, which varied in their precise location, suggesting that the ventilatory and arousal responses were due to separate populations of glutamatergic neurons in the lateral PB region^20^. We later found that a subset of these neurons in the external lateral parabrachial nucleus that express the peptide CGRP (PBel^CGRP^ neurons) play a critical role in transmitting the arousal signal to cause EEG desynchronization in response to hypercapnia, with minimal effect on respiration^19^. In addition, we observed a second population of non-CGRP neurons adjacent to the PBel^CGRP^ group in the central lateral, lateral crescent and Kölliker-Fuse parabrachial subnuclei that are activated in response to CO2 exposure in mice^23–31^. Recent studies showed that neurons in the lateral PB expressing the transcription factor Fork head Box protein 2 (FoxP2)^32–34^ have a distribution that is complementary to the PBel^CGRP^ neurons including the regions containing the non-CGRP neurons that express cFos after CO2 exposure and which project to premotor and motor neurons that innervate the diaphragm and genioglossus muscles^35^. Therefore, we hypothesized that whereas the PB^CGRP^ neurons wake up the forebrain during apneas, the adjacent PB^FoxP2^ neurons may trigger activation of ventilatory effort during apnea.

To test this hypothesis, we examined the role of the PB^FoxP2^ neurons in respiration and arousal response to CO2. We started by investigating their response (cFos expression) to CO2 exposure as well as in vivo recording of PB^Foxp2^ neuronal activity by using intracellular calcium imaging during spontaneous sleep-wake and during exposure to CO2. We also investigated their contribution to the respiratory changes by optogenetically photo-activating PB^FoxP2^ neurons at different frequencies during either normocapnia or hypercapnia and by optogenetically photo-inhibiting them using archaerhodopsin T (ArchT), while mice were exposed to a CO2 stimulus.

## Results

### Activation of the PB^FoxP2^ neurons by CO2:

#### cFos expression in the PB^FoxP2^ neurons:

**Figure 1:**
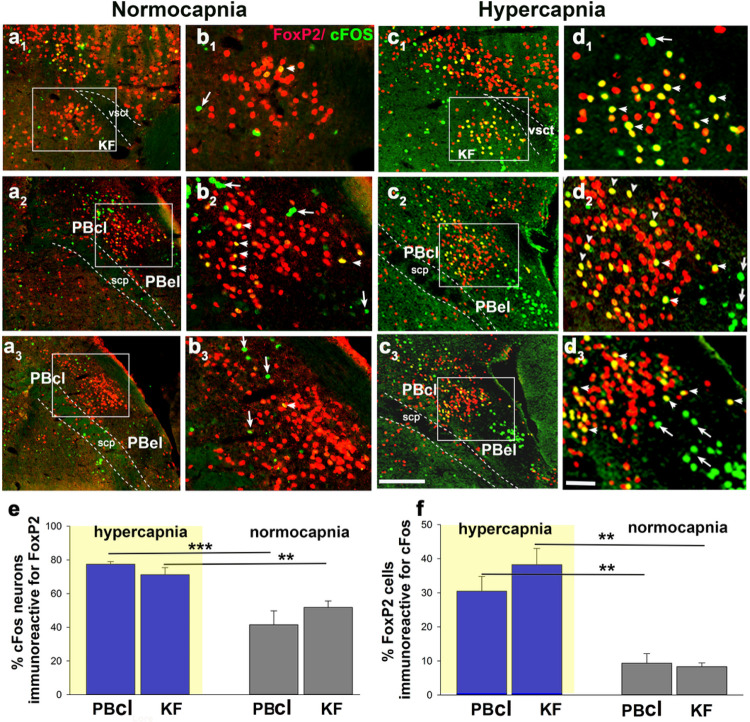
CO2 activates cFos expression in the PB^FoxP2^ neurons: Photomicrographs in columns **a-d** represent sections at the rostral (level 1, first row), middle (level 2, second row) and more caudal (level 3, third row) portions of the PB labeled immunohistochemically for both the immediate early gene cFos (green) and FoxP2 (red), from a mouse that was exposed to 2h of either normocapnic room air (columns **a and b**) or 10% CO2 (columns **c and d**). The insets in **a** and **c** demarcate the areas that are magnified in the **b** and **d**. Double-labeling (yellow nuclei) was prominent in the KF and in the central lateral FoxP2 clusters in mice exposed to CO2 but not those breathing normocapnic air. The arrow heads in **b** and **d** point to doubly-labeled neurons, while the arrows mark neurons that were only labeled for cFos (green), a large cluster of which represent the CGRP neurons in the PBel (**c2, c3**). The graphs in e and f compare the percentage of the cFos cells that also expressed FoxP2 (**e**), and the percentage of Foxp2 cells that were also labeled for cFos (**f**) in the PBcl and KF areas, after exposure to 10% CO2 (hypercapnia, n=5) or room air (normocapnia, n=3). The groups were analyzed using a one-way ANOVA, where ***- P<0.001; **- P<0.01. Scale in c= 200μm; d= 20 μm. Abbreviations: KF- Kölliker Fuse PB subnucleus; PBcl- central lateral PB subnucleus; PBel- external lateral PB subnucleus; scp- superior cerebellar peduncle; vsct- ventral spino-cerebellar tract.

We subjected mice to either 2h of 10% CO2 (10%, CO2, 21% O2, 69% N2; n = 5) or normocapnic air (room air: 21% O2, 79% N2; n = 3) and then examined brain sections using immunohistochemistry for both FoxP2 and cFos, an immediate early gene, used as a functional marker for activity in neurons. Because both cFos and FoxP2 are transcription factors, they are localized to the nucleus of neurons (Fig. 1). Exposure to CO2 caused a large increase in number of FoxP2 neurons that also showed cFos expression in the KF (38.3 ± 5%) compared to room-air (8 ± 1%; F_1,7_= 21.88, P = 0.003; Fig. a1–d1 and f). The doubly labeled KF neurons formed a cluster located medial to the ventral spinocerebellar tract along the ventrolateral surface of the rostral PB. Slightly more caudally in the lateral PB, large numbers of neurons that showed cFos activation in response to CO2 exposure were found in the PBel (non-FoxP2 neurons, in the region of the CGRP neurons) and in the central lateral PB, just dorsal to the CGRP cluster and wrapping around its lateral margin. 49 ± 5% of the total cFos immunoreactive neurons in the PBL were FoxP2 neurons (F_1,7_= 11.95, P = 0.013; Fig. a2–d3 and f). After CO2 exposure, the majority of the cFos + neurons in both the PBcl (78 ± 2%) and in the KF (71 ± 4%) showed FoxP2 expression (PB- F_1,7_= 31.85, P = 0.001; KF- F_1,7_= 9.8, P = 0.02) compared to the 42 ± 8% in PBcl and 52 ± 4% in KF of the room-air group (Fig. 1e). Of the total population of FoxP2-expressing neurons in the PBcl (PB^FoxP2^ neurons) 30 ± 4% expressed cFos after CO2 exposure compared to 9 ± 3% in the control mice (room-air). Therefore, the PB^FoxP2^ and KF^FoxP2^ neurons constitute the bulk of the non-CGRP neurons in the region adjacent to the PBel^CGRP^ neurons that were also responsive to CO2. Interestingly, we found a small number of KF^FoxP2^ neurons that express the gene for CGRP (*Calca*) (supplementary Fig. 1), by using florescence in situ hybridization for CGRP in FoxP2-L10 mice. The FoxP2^+^ CGRP neurons in the KF (mean diameter 18 ± 0.7 μm) tend to be significantly smaller (F_1, 18_= 72.4; P < 0.001) than the ones that are not FoxP2^+^ (26.3 ± 0.7 μm).

#### In vivo measurement of PB^FoxP2^ neuronal activity by fiber photometry:

**Figure 2:**
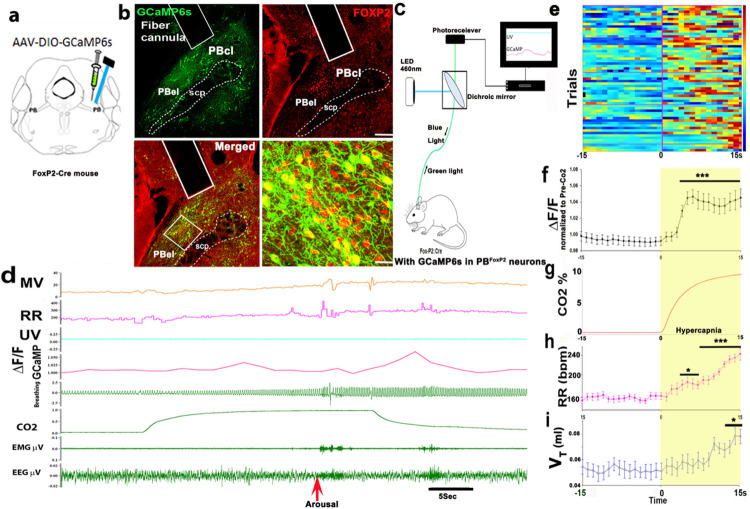
In vivo intracellular calcium imaging of PB^FoxP2^ neurons by fiber photometry during exposure to CO2: *A*deno-associated virus (AAV) expressing Cre-dependent GCaMP6s was injected into the lateral PB of FoxP2-Cre mice (**a**), resulting in green GCaMP6s expression in the PB^FoxP2^ neurons (red) (**b**). The track of the implanted optical fiber just dorsal to GCaMP-expressing PB^FoxP2^ neurons is outlined in b and allowed us to record their calcium activity as shown in the schematic in **c**. A representative fiber photometry recording (**d**) shows the activity profile of the PB^FoxP2^ neurons (**ΔF/F**), with simultaneous EEG/EMG signals and the respiratory rate (**RR**) and minute ventilation (**MV** = RR × tidal volume, V_T_), both of which increased significantly in each CO2 trial. The control UV signal (isobestic at 405nm) was also recorded. Note that the GCaMP signal increases slowly during CO2 exposure, but more sharply as the animal wakes up (abrupt change in EMG and EEG about 25 sec after CO2 onset- marked by red arrow). The ΔF/F from 59 trials from n=5 mice is depicted in **e** as a heat-map illustrating activity 15 s before and after onset of CO2 exposure. The graphs below show the mean + SEM ΔF/F normalized to the pre-CO2 values (**f**), % CO2 in the plethysmograph (**g**), respiratory rate (RR, **h**) and tidal volume (V_T_, i) across the same trials. Two way ANOVA compared the changes in ΔF/F, RR and V_T_ during CO2 exposure compared to the pre-CO2 baseline, where ***- P<0.001; **- P<0.01 and *- P<0.05. Scale in b, 200μm (lower left) and 20μm (lower right). Scale in b= top right 100μm; bottom right= 20 μm. Abbreviations: PBcl- central lateral PB subnucleus; PBel- external lateral PB subnucleus; scp- superior cerebellar peduncle.

To capture and analyze the pattern of activation of the PB^FoxP2^ neurons by CO2 *in vivo*, we injected an adeno-associated viral vector (AAV) expressing Cre-dependent GCaMP6s into the lateral PB of *Foxp2*^*tm1.1(cre)Rpa*^/J (FoxP2-Cre) transgenic mice (developed by Dr. Richard Palmiter at University of Washington, and donated to the Jackson Laboratory)^34,36^. We double-labeled sections through the PB from FoxP2-Cre::L10 reporter mice (in which FoxP2 cell bodies are marked by GFP) with FoxP2 immunohistochemistry (which stained nuclei of FoxP2 expressing neurons magenta) to verify that Cre-recombinase was expressed eutopically in FoxP2 expressing neurons (supplementary Fig. 2a-d). All of the neurons transfected by an AAV that expressed Cre-dependent GCaMP6s (which also fluoresces green) also showed Foxp2 expression in their nuclei (red in Fig. 2b). The implanted glass fiber targeting the GCaMP expressing PB^FoxP2^ neurons allowed us to record intracellular calcium levels, a surrogate for neuronal activity (Fig. 2c). The population activity of the PB^FoxP2^ neurons was then correlated to the identified sleep-wake states, respiration and their changes in response to repetitive CO2 exposures.

In n = 5 mice, optical fibers were located in proximity to the GCaMP expressing PB^FoxP2^ neurons (Fig. 2b). A representative example of a fiber photometry recording across an exposure to CO2 is illustrated in Fig. 2d. In these mice, GCaMP fluorescence increased when the mouse transitioned from NREM sleep to either wake or REM sleep state (supplementary Fig. 3a and b). The increased activity of PB^FoxP2^ neurons when emerging from NREM sleep may contribute to the sudden increase in respiratory effort when an animal exposed to CO2 awakens from NREM sleep.

All trials with 30 sec exposures to 10% CO2 also resulted in cortical arousal in the latter half of the trial. Therefore, to avoid the confounding effects of significantly higher GCaMP responses caused by transition to wakefulness, we chose to analyze only the first 15s of the trial segments, which in all cases preceded cortical arousal. Although the actual level of fluorescence during exposure to CO2 waxed and waned during individual exposures (see heatmaps in Fig. 2e), when the level of ΔF/F (change in GCaMP activity/background fluorescence) was summated across trials (Fig. 2f) and over 15s prior to and during CO2 in all trials, there was a robust and statistically significant increase in fluorescence of FoxP2 neurons during exposure to CO2 (F_1, 1682_= 62.2; P < 0.001) (Fig. 2f, ΔF/F normalized to pre-CO2), with the fluorescence peak for each of the 59 trials during CO2 exposure increasing by 6.4 ± 0.9% (F_1, 116_= 45.95 P < 0.001) compared to pre-CO2. This increase in ΔF/F was also accompanied by an increase in respiratory efforts in response to CO2 as measured in 59 trials from 5 mice (Fig. 2g–i). During this period we found a significant increase in respiratory rate (RR, 40 ± 1% increase for the last 5 breaths of the 15 sec interval after onset of CO2 exposure compared to the last 5 breaths prior to onset of CO2; F_1, 1662_= 5.7, P < 0.001, Fig. 2h), and a smaller increase in tidal volume (V_T_) that lagged behind the increase in RR (Fig. 2 h–i) but was statistically significant (28 ± 0.89% increase) for the last three breaths of the 15 sec interval (P = 0.025; P = 0.004 and P = 0.037) (Fig. 2i). The GCaMP fluorescence (ΔF/F) is also shown as heat-maps for all the trials for 15 s before and after CO2 exposure (Fig. 2e). Analysis of this data shows that the average latency of the increase in intracellular calcium (Ca_i_) to its first peak was 9.6 ± 0.57 sec, and in only 13.5% of the trials was the latency less than 5 sec (Fig. 2f). This observation aligns with the rise in RR starting at about 5 sec (approximate time that it takes for CO2 levels to rise in the chamber and then reach the circulation). The RR increased progressively in all trials for at least the next 10 sec (Fig. 2h), and then increased abruptly at the time of cortical arousal, which occurred between 15 and 30 sec in all trials.

**Figure 3:**
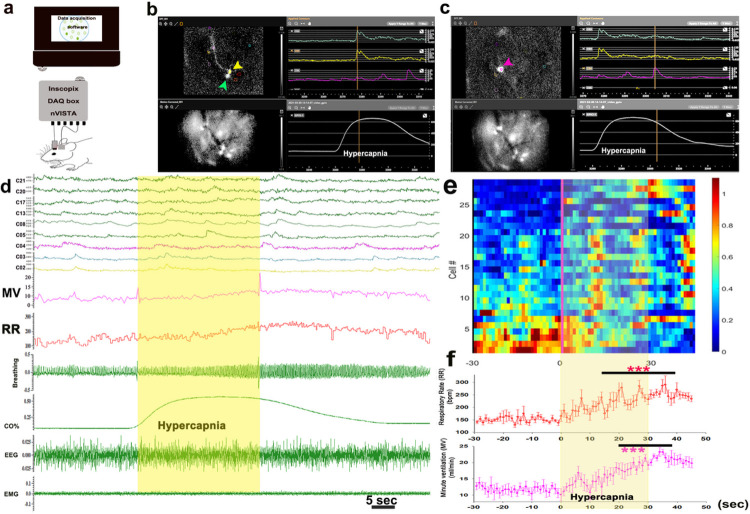
In-vivo activity of individual PB^FoxP2^ neurons during CO2 exposure: Gradient-index (GRIN) lens were implanted above the injection sites in the PB of FoxP2-cre mice injected with Cre-dependent AAV-GCaMP6s (**a**) and the calcium activity profiles (ΔF/F) of individual PB^Foxp2^ neurons in response to the CO2 was acquired. Representative intracellular calcium activity of 3 neurons is shown for 30 sec before and 60 sec after onset of CO2 (**b**) and in the same trial for 10 sec before and 60 sec after onset of CO2 (c). Two cells (marked by green and yellow arrowheads whose activity profiles are also plotted in green and yellow) shown in **b** had fluorescence that peaked at about 17–19 sec after exposure, before the maximal changes in respiration. A third cell (marked by a magenta arrow and activity profile) peaked roughly 50 sec after onset of the CO2 trial (**c**), but while CO2 levels were still high. A representative trial that showed no cortical arousal during CO2 exposure but had a clear respiratory response to the 8% CO2 as seen by a gradual rise in the respiratory (RR) and the minute ventilation (MV) is shown in **d**. The ΔF/F from 9 cells is also plotted, showing the overall increase in intracellular calcium across the population, with most cells showing peaks at the time of maximal respiration, but with substantial variability across neurons (**d**). A heat map of the mean ΔF/F over 4–7 trials (during which animals exposed to 8% CO2 did not awaken) is shown for all 28 cells in **e** (blue to red- shows low to high ΔF/F). Note that although the RR and MV summated over these trials increased relatively smoothly (**f**), the activation of the PB^FoxP2^ neurons occurred in waves, and of the 17 cells that showed 3 peaks, 10 of them showed second and third peaks that were 17–19 sec apart and were synchronous in time after CO2 exposure, but that not every neuron participated in each wave of excitation. Two way ANOVA compared the changes in RR and MV post CO2 to the Pre-Co2 baseline, where ***- P<0.001.

We then analyzed the neuronal calcium activity of 28 neurons for the periods where the mice (n = 3) were exposed to CO2 (Fig. 3b and c) in a plethysmograph that allowed us simultaneously to record their breathing as well as EEG and EMG. For this analysis, we wanted to prevent confounds introduced by cortical arousal (EEG desynchronization and increase in motor activity), so we reduced the CO2 concentration to 8% and only used trials that showed no cortical arousal during 30s of CO2 exposure and in the subsequent 15 sec when CO2 levels in the plethysmograph were still high (see Fig. 3d, also video-CO2 responsive neurons.mp4). In these experiments 8% CO2, caused a 65 ± 4.4% increase in RR (F_1, 530_= 4.95, P < 0.001, pre vs. post) to 249 ± 5 bpm 20–40sec after CO2, compared to 152 ± 2 bpm at pre-CO2 levels. Minute ventilation also increased significantly (MV, F_1, 530_= 5.8, P < 0.001) with a 87.5 ± 4.4% increase 20–40 sec after CO2 onset (22.3 ± 0.4 ml/min) compared to pre-CO2 levels of 12 ± 0.26 ml/min (Fig. 3d, f). V_T_ showed a smaller but still significant increase (F_1, 360_= 1.53; P = 0.043) by 28 ± 5% during 20–40sec after CO2 onset (0.094 ± 0.002ml) compared to 0.074 ± 0.0016ml at pre-CO2. An example of simultaneous neuronal activity profiles for 9 cells during one representative CO2 trial is shown in Fig. 3d. We analyzed the activity of all n = 28 cells, for 30s before and for 90s after the CO2 stimulus in 4–7 trials during which the animals remained in NREM sleep for this period of time, and plotted the normalized fluorescence (ΔF/F) as a heat map (Fig. 3e). A small number of cells (4/28, 14%) were more active prior to the CO2 exposure, and were less active during the exposure. We termed these CO2-Off cells. The remaining 24 CO2-On neurons all showed periodic increases in Ca_i_ after exposure to CO2, with 17/24 cells showing three distinct fluorescence peaks during and after CO2 exposure. 23/24 cells showed a first peak in activity with a mean latency of 11 ± 0.6 sec after onset of the CO2, 21/24 showed a second peak at a mean of 27 ± 1.6 sec, and then 17/24 at 41 ± 2.8 sec; 1/24 only peaked at 33s after CO2 onset (Fig. 3f). Of the 17 cells that showed all three peaks, 10 cells peaked first at 11 ± 0.21 sec after CO2 onset and then showed periodicity of 17–19 sec for appearance of the second and third peak. Smaller numbers of neurons (10–12) also showed peaks in activity at roughly 60 and 75 sec. None of these neurons displayed this pattern of periodic and synchronized activity during NREM sleep prior to CO2 exposure, although the periodic waves of activity were similar to those we observed in individual PB^FoxP2^ neurons in animals breathing room air during wake and REM sleep. These data suggest that PB^FoxP2^ neurons may have a tendency to be activated in periodic waves that are synchronous across the population during wake and REM, but that these waves of activity may be suppressed during NREM sleep. Exposure to CO2 may then remove this suppression, allowing resumption of periodic, synchrous waves of activity with a periodicity during CO2 exposure in NREM sleep of about 15 sec. Whether these synchronous waves of activity originate from a rhythmic pattern of input, or are generated by recurrent activity in a local network or even are a cell autonomous response is not clear.

### Photo-activation of PB^FoxP2^ neurons and respiration:

**Figure 4:**
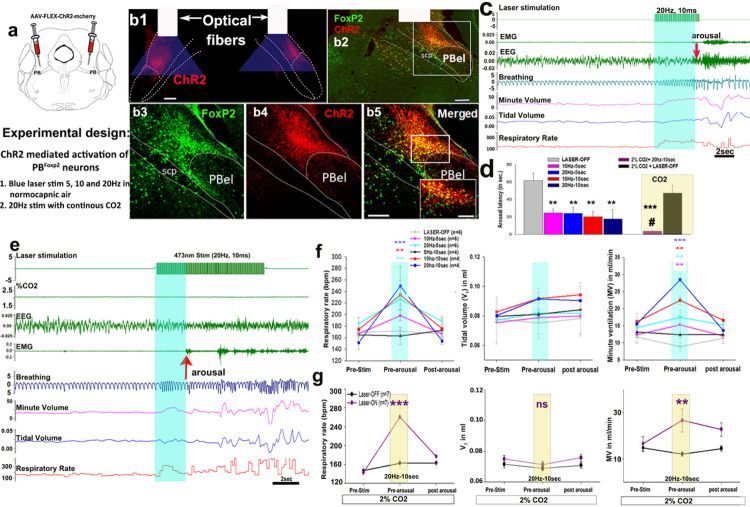
Effect of photoactivation of PB^FoxP2^ neurons on respiration: FoxP2-cre mice were injected bilaterally targeting the PB with cre-dependent AAV-Flex-ChR2-mCherry (red **a, b**; also immunostained for FoxP2 (green florescence, **b2,3,5**) and implanted for EEG/EMG recording and with bilateral glass optical fibers to target illumination of the ChR2-expressing FoxP2 neurons (**b1,2**). The right side of the coronal section in b1, is also shown in b2 with immune-labeling for Foxp2 (green). The area within the box in **b2** is shown at higher magnification in **b3–b5**, and the doubly labeled neurons (yellow) are shown at higher magnification in the inset in **b5** (scale= 50 μm). Scale in b1- b5=100μm. Abbreviations: PBel- external lateral PB subnucleus; scp- superior cerebellar peduncle. Respiration was analyzed for 5 breaths pre-stimulation, then for 5 breaths just before the end of stimulation or before cortical arousal and for 5 breaths after cortical arousal when stable breathing was attained without EMG artifacts. A representative trial with stimulation at 20 Hz (in normocapnic air) showed gradually increasing respiration which preceded the cortical arousal that occurred at 4.5 sec in this trial (**c**). In trials with 5 sec stimulation, the animals on an average woke up around 15 sec after the stimulation stopped (**d**), suggesting that the awakening was not due to the stimulation of the PB^FoxP2^ themselves, but may have been elicited by some aspect of the response to stimulation (e.g., respiratory efforts). Trials with stimulation for 10 sec usually caused EEG arousal either just before or after the termination of stimulation. Arousal latency was decreased dramatically (by 83%) by exposing the mice to continuous 2% CO2 during opto-stimulation (**d, shown in yellow rectangle**). A representative trial of stimulation at 20Hz for 10sec with continuous 2% CO2 is shown in **e**. Graphs in **f** compare the RR, V_T_ and MV in mice subjected to laser stimulation at 5, 10 or 20Hz vs. no laser (Laser-OFF) for either 5 or 10s during normocapnia. Graphs in **g** compare the RR, V_T_ and MV parameters in the mice subjected to laser stimulation at 20Hz vs. no laser (Laser-OFF) for 10s, in mice continuously exposed to 2% CO2 (as shown in **e**). The average values (mean ± SEM) are plotted for 5 breaths before onset of stimulation, during stimulation but before cortical arousal and in the post stimulation period after the cortical arousal when stable breathing is attained without EMG artifacts (**e-g**). Two-way ANOVA was used for statistical comparison, where **= P<0.01 (vs. pre-stimulation/Laser OFF); ***= P<0.001 (vs. pre-stimulation/Laser OFF), **#**= P<0.05 (vs. Laser-ON at normocapnia).

To test the effect of activation of PB^FoxP2^ cells on respiration, we injected the PB of FoxP2-cre mice (n = 10) bilaterally with cre-dependent AAV-Flex-ChR2-mCherry (Fig. 4a), and implanted them for EEG/EMG recording along with bilateral optical fibers to target illumination of the ChR2 expressing FoxP2 neurons in the PB (Fig. 4b). We recorded animals for sleep and breathing in normocapnic room air and during activation of ChR2 with a blue laser (473nm) using 10ms pulses at 5, 10 or 20Hz, for either 5s or 10s every 5 minutes. Trials in which mice were in NREM sleep for at least 30s before stimulation were analyzed for the effect of optostimulation on respiration. In 6 out of 10 implanted mice, the optical fibers accurately targeted the ChR2 expressing PB^FoxP2^ neurons as shown in Fig. 4b. These animals consistently showed respiratory responses during optostimulation but also had EEG arousal either late in the stimulation or shortly after offset (Fig. 4c, d) in 70% of the trials. In the four cases where fiber implants were placed either medial or lateral to the PB^FoxP2^ ChR2 expressing neurons, neither 10 nor 20Hz stimulation produced any effect on respiration or cortical arousals. To distinguish the increment in ventilation due to waking, we measured RR and V_T_ for 5 breaths pre-stimulation, 5 breaths just before the end of stimulation (or just before onset of arousal, whichever occurred first) and 5 breaths after the cortical arousal.

While breathing normocapnic air, 10Hz stimulation progressively increased RR (18.5% with 5 sec and 42% with 10 sec stimulation, P = 0.013; F_2, 63_= 19.75, P < 0.001) and minute ventilation (MV) (17.9% with 5 sec and 26% with 10 sec stimulation, for 5 sec - P = 0.03; for 10 sec- P < 0.001; F_2, 63_= 10.43; P < 0.001) compared to the pre-stimulation period, but had little effect on tidal volume (F_2, 63_= 0.35; P = 0.71). Stimulation at 20 Hz increased RR by 35% at 5 sec vs. 69% at 10 sec (F_1, 106_= 122.9, P < 0.001) and MV by 25% at 5 sec and 40% at 10 sec (Fig. 4f). This pattern was consistent with the fiber photometry results, in which RR began to increase at the same time as calcium activity of PB^FoxP2^ neurons, but increases in V_T_ lagged by about 10 sec. However, no significant changes on respiration were observed with photo-stimulations at 5 Hz (Fig. 4f).

To explore the interaction between optogenetic stimulation and activation of PB^FoxP2^ neurons due to elevated CO2, we repeated the 20Hz, 10 sec stimulation in mice breathing 2% CO2 (Fig. 4e, representative example). First, we observed that the presence of 2% CO2 throughout the experiments dramatically decreased the latency of awakening to the laser stimulus to 2.22 ± 0.3 sec compared to 21.7 ± 1.7 sec in normocapnic air (F_4, 27_= 3.2; P = 0.031) (Fig. 4d). Despite the relatively brief period of laser stimulation before awakening in 2% CO2, we also found a large and almost immediate increase in RR (81%; F_2, 12_= 44.25; P < 0.001) and MV (38%; F_2, 12_= 8.08; P = 0.006), with no change in V_T_ (Fig. 4g), similar to that found with stimulation in normocapnic air (Fig. 4f). Interestingly, the EEG arousal occurred at around the same RR as with CO2 or optogenetic stimulation alone (i.e., approximately 240–250 bpm), raising the possibility that the arousal caused by stimulation of the PB^FoxP2^ neurons may be due to the sensory feedback from the increased ventilatory effort, rather than being a primary effect of the PB^FoxP2^ neurons.

**Figure 5:**
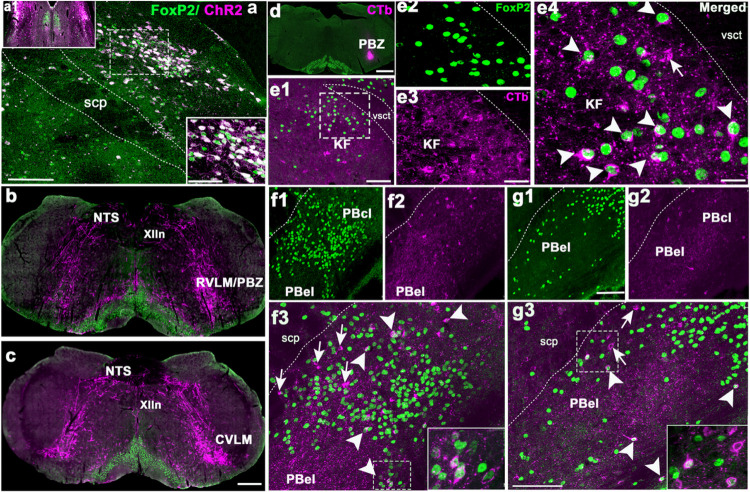
Descending projections of the PB^FoxP2^ neurons: Bilateral injections of Cre-dependent AAV-ChR2 (magenta) into the PB in a FoxP2-Cre mouse (**a1**) is shown with immuno-labeled for FoxP2 (green); nearly all of the ChR2-labeled cell bodies are doubly labeled (white) in the area along the dorsal margin of the PBel (**a**, magnified view of injection site on the right side). The inset in a, is a magnified view of the area marked by the dashed-rectangle. Descending fibers and terminals in the medulla are shown in magenta in **b** and **c**. Note that inferior olive neurons (green) also express FoxP2. Scale in a= 100μm, in c=500μm. The photomicrograph in **d** shows the injection site of the retrograde tracer cholera toxin subunit b (CTb −0.2%, magenta) in the pre-Bötzinger area (PBZ, **d**) (n=3; scale= 500μm). The injection of CTb in PBZ (**d**) retrogradely labeled many FoxP2 neurons in the KF (**e1–e4**) and the PB area (**f1–g3**), especially in the lateral crescent and the central lateral PB (PBcl) areas that surround the PBel subnucleus. The arrow heads in e4, f3 and g3 mark the cells doubly labeled for FoxP2 and CTb (white), while the arrows mark cells labeled only with CTb (magenta). The insets in f3 and g3 are 2x magnified views of the areas encompassed by dashed rectangles, highlighting the double labeled cells. Scale in e1= 100 μm; in e3= 50 μm and in e4= 30 μm. Scale in g1 and g3= 100 μm. Abbreviations: CVLM- caudal ventrolateral medulla; KF- Kölliker Fuse PB subnucleus; NTS, nucleus tractus solitarii; PBZ- pre-Bötzinger area; PBcl- central lateral PB subnucleus; PBel- external lateral PB subnucleus; RVLM – rostral ventrolateral medulla; scp- superior cerebellar peduncle; vsct- ventral spino-cerebellar tract; XIIn – hypoglossal nucleus.

Further, we used the mice from these experiments (n = 6) to map the projections of the ChR2-expressing PB^FoxP2^ neurons (Fig. 5a). We found extensive mCherry labeling of fibers and terminals in the pre-Bötzinger complex (PBZ) and caudal ventrolateral medulla (CVL), and more moderate numbers of labeled terminals in the nucleus of the solitary tract (NTS) and hypoglossal nucleus (XIIn) (Fig. 5 b and c). To confirm these projections we also injected a retrograde tracer CTb in the PBZ area (n = 3; Fig. 5d) and mapped the retrogradely labeled FoxP2 neurons in the PB and KF. Doubly labeled neurons were found in the KF (Fig. 5e1–e3), the central lateral PB subnucleus and in the lateral crescent zone along the lateral edge of the external lateral PB connecting the two major clusters (Fig. 5 f–g). Thus the PB^FoxP2^ population projects to medullary targets (Fig. 5) that are consistent with mediating CO2-evoked changes in ventilation. PB^FoxP2^ neurons also showed ascending projections to the median and lateral preoptic area and ventral to the zona incerta in the lateral hypothalamus, but do not project to the substantia innominata in the basal forebrain and the central nucleus of the amygdala, which are key targets of the PB^CGRP^ neurons mediating CO2-induced EEG arousal.

### Photo-inhibition of PB^FoxP2^ neurons during exposure to CO2:

**Figure 6:**
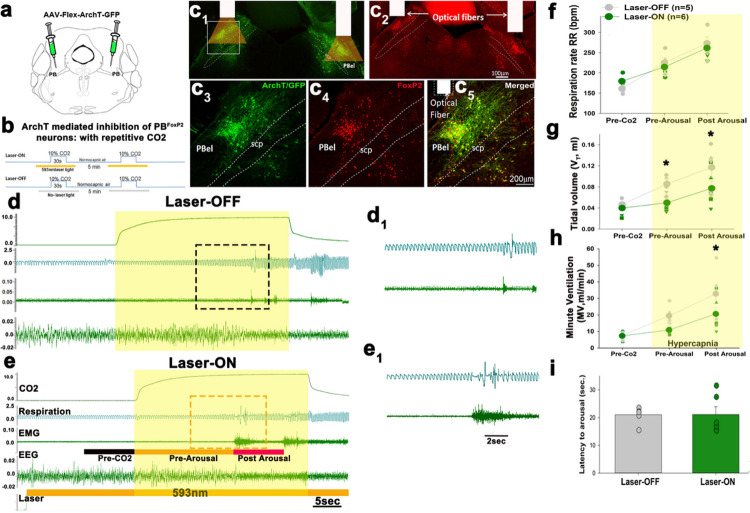
Acute optogenetic silencing of the PB^FoxP2^neurons blocks hypercapnia-induced respiratory drive: FoxP2-Cre mice were injected with AAV-FLEX-ArchT (**a**), and implanted for EEG/EMG and with bilateral optical fibers targeting PB^FoxP2^ neurons (**c1, 2**). There was virtually complete co-expresion of FoxP2 (red) and ArchT (green, **c3–5**). The experimental strategy to test respiratory responses to 10% CO2 given for 30s every 300s, with and without photo-inhibition of the PB^FoxP2^ neurons is shown as schematic in **b**. Representative examples of a laser-ON and a Laser-OFF trial are shown in **d** and **e**, with magnified views of the bounding box from each trial shown in **d**_**1**_ and **e**_**1**_. For each stimulation, the laser was switched ON for 60s beginning 20 sec prior and extended for 10 sec after the CO2 stimulus (30 sec, illustrated by yellow box) as shown in **e**. Photoinhibition of PB^FoxP2^ neurons significantly reduced the increases in V_T_ (**g**) and MV (**h**) but not RR (**f**) caused by CO2 exposure. Two way ANOVA, with *- P<0.05, compared to the pre-stimulation. The photo-inhibition of PB^FoxP2^ neurons did not change the latency to arousal (**i**).

To test the necessity of PB^FoxP2^ neurons in producing the respiratory responses to CO2 during sleep, we photo-inhibited them during hypercapnia. FoxP2-cre mice were injected in the ventral part of the LPB with AAV-Flex-ArchT (n = 12) (Fig. 6a) and implanted with EEG/EMG electrodes and bilateral optical fibers targeting the PB^FoxP2^ neurons (Fig. 6c1–c5). We analyzed the respiratory responses to 10% CO2 given for 30s every 300s, with and without photo-inhibition of the PB^FoxP2^ neurons using a 593 nm laser light (Fig. 6b). The laser light was on for 60s, beginning 20s prior and extending to 10s after the CO2 stimulus (30s) (representative trials of CO2 exposure are shown in Fig. 6 d and e).

Photoinhibition of PB^FoxP2^ neurons significantly reduced the increase in V_T_ caused by CO2 (F_2, 27_= 9.67; P = 0.004) by 42% (p =−0.027) pre-arousal and by 34% (p = 0.015) after arousal. It also reduced the increase in MV caused by increased CO2 (F_2, 27_= 6.25; P = 0.017) by 37% post-arousal (P = 0.015), but the reduction pre-arousal was not statistically significant (p = 0.081) (Fig. 6g, h). Photoinhibition of PB^FoxP2^ neurons had little effect on the RR increases induced by CO2 (Fig. 6f) and no effect on latency to arousal.

## Discussion

Our previous work identified a population of CGRP neurons in the PBel that project to the forebrain and are required for arousal from sleep during CO2 exposure, but make little contribution to respiratory efforts. Here we report a second adjacent population of neurons that is marked by expression of the FoxP2 transcription factor and is required for normal respiratory response to CO2 during sleep, but not arousal. These PB^FoxP2^ neurons are located in clusters in the PBcl just dorsal to the CGRP group and in the KF subnucleus, and along a narrow bridge just lateral to the CGRP group (the lateral crescent) connecting the two major clusters. This population of PB^FoxP2^ neurons projects intensely to medullary respiratory areas as well as to hypothalamic targets (lateral hypothalamus, preoptic area) associated with regulation of wake-sleep. However, optogenetic stimulation of the PB^FoxP2^ neurons causes immediate increases in ventilation, but only delayed and inconsistent arousals, and while photoinhibition reduces the ventilatory increases caused by CO2 it does not affect arousal. Hence, we interpret the PB^FoxP2^ neurons as primarily mediating the ventilatory response to CO2.

We further found that optogenetically activating the PB^FoxP2^ neurons eventually also wakes the animal up, although often after the PB^FoxP2^ neurons are no longer being stimulated, and at levels of ventilatory effort that are similar to that at the time of arousal in an animal exposed to 10% CO2. We interpret these findings as an indication that CO2 exposure largely and independently activates populations of PB^CGRP^ neurons that cause EEG arousal and PB^FoxP2^ neurons that cause increased ventilation. However if animals are forced to increase ventilation enough, they are awakened by the effort, and if they wake up during CO2 exposure, they further increase ventilation. This relationship underscores the synergy between the behavioral and respiratory motor responses to insure survival when an animal is either apneic or asphyxiated.

We used three different intersecting strategies to identify the role of the PB^FoxP2^ in the ventilatory response to CO2 during sleep. First, we showed that among the neurons in the PBcl and KF (i.e., outside the PBel^CGRP^ group) that show cFos expression after CO2 exposure, nearly 75% express FoxP2. This PB^FoxP2^ population had no overlap with the PBel^CGRP^ neurons (i.e., no PBel^CGRP^ neurons expressed FoxP2), indicating that they are independent cell populations. Interestingly, we found a small number of KF neurons that are CGRP-immunoreactive (supplementary Fig. 1). These neurons are a bit smaller than those in the PBel, and as shown by Geerling and colleagues^34^, they project to respiratory sites in the medulla, like the remainder of KF glutamatergic neurons (see also Yokota et al., 2015^35^). We had missed this population in our previous reports because they appear to express CGRP at a lower level than the larger PBel^CGRP^ neurons (Nardone, Saper and Lowell, in preparation)^37^. We use Calca-ires-Cre-ER mice in our experiments for identifying and manipulating CGRP neurons, and apparently the level of Cre expression in these small KF CGRP neurons was not sufficient to cause recombination events. By contrast these neurons appear to have greater Cre expression in the Calca-Cre knockin mice used by Geerling et al., and so are more readily identified. Interestingly, our in situ hybridization results reported here (supplementary Fig. 1) indicate that at least some of the KF^CGRP^ neurons also express FoxP2, underscoring that they are a separate population from the PBel^CGRP^ neurons and demonstrating the value of FoxP2 as a marker for identifying the neurons that drive ventilatory responses to CO2. It should be pointed out, however, that FoxP2 is expressed more widely in more dorsally located neurons in the lateral PB, but only the FoxP2 neurons in the PBcl, KF and lateral crescent express cFos in response to CO2. We are currently searching for more selective markers for this neuronal population.

The second strategy we used to investigate the relationship of the PB^FoxP2^ neurons with the ventilatory response to CO2 was the employment of fiber photometry to study the intracellular calcium responses of PB^FoxP2^ neurons during wake-sleep and during CO2 exposure. The PB^FoxP2^ neurons were clearly wake and REM active. This finding is consistent with the general reduction of respiration during NREM sleep, and the fact that arousal from NREM sleep caused a rapid increase in both ventilation and in the calcium signals from the PB^FoxP2^ neurons. This population of neurons showed a brisk increase in calcium signal beginning a few seconds after onset of a 10% CO2 stimulus, but persisting for many seconds after the stimulus was removed. We then resolved the responses of individual PB^FoxP2^ neurons with GCaMP6s endoscopy during exposure to 8% CO2 (a concentration chosen because it generally did not wake up the animals during a 30 sec exposure). Surprisingly, the individual PB^FoxP2^ neurons showed multiple recurrent peaks of calcium activation, with the first peak at 11.0 ± 0.2 s after onset of the gas and then further peaks at roughly 17–19 sec intervals. The mechanism for these rhythmic pulses of activity in the PB^FoxP2^ neurons remains a mystery. These experiments admittedly involved only a small number of PB^FoxP2^ neurons and because of the limited depth of field of the GRIN lens we used, all of them were in a small cluster near the surface of the lens. However, if the larger population of PB^FoxP2^ neurons also responds rhythmically to CO2, our results would suggest that the much smoother population response as imaged by fiber photometry is probably a summation of the activity of different subsets of PB^FoxP2^ neurons that are activated at different times in different locations in the PBcl and KF during CO2 stimulation. Our anterograde and retrograde tracing experiments indicate that PB^Foxp2^ neurons may mediate these effects on respiration by their descending projections to the medullary targets involved in ventilatory rate and volume^35^.

The third strategy we used to characterize the role of the PB^FoxP2^ neurons in the response to CO2 involved optogenetic excitation and inhibition. When the PB^FoxP2^ neurons were opto-stimulated at 10 or 20Hz while mice were breathing normocapnic air, they showed a statistically significant increase in RR and MV (but not V_T_) that was greater both with higher frequency or prolonged duration of stimulation.

Interestingly, the animals only awakened either late in the stimulation episode or immediately afterward, suggesting that the EEG arousal was not a direct effect of the stimulation. In this regard, it was interesting that when animals were stimulated optogenetically while breathing 2% CO2, the latency of the arousal was shorter, but occurred at about the same RR as the opto-stimulation while breathing room air or in response to CO2 without optostimulation (i.e., awakening in all three conditions occurred at about 240–250 breaths/min, or when RR was about 50% greater than baseline). This observation suggests that the arousal may have been due to somatomotor feedback from the increased ventilatory effort. Alternatively, it is possible that either the PB^FoxP2^ neurons or brainstem cell groups downstream from them may have collaterals that activate PBel^CGRP^ or some other arousal neurons. It would be useful to test whether simultaneous suppression of PBel^CGRP^neurons while activating PB^FoxP2^neurons might permit increased ventilation without EEG arousal.

**Figure 7:**
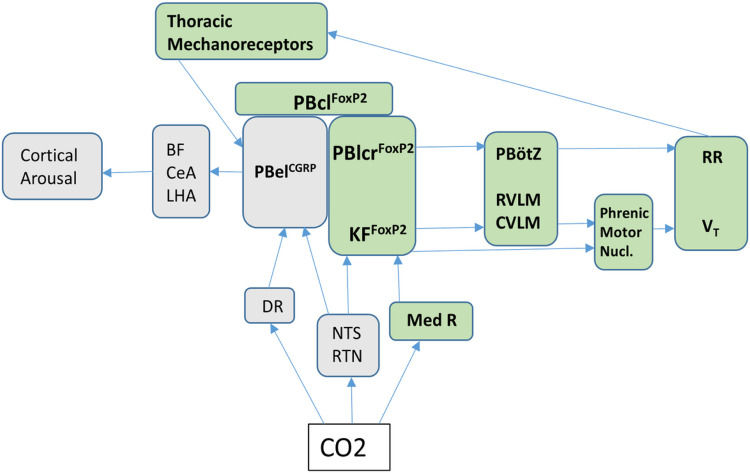
Neural circuit for mediation of hypercapnia induced increase in ventilation: PB neurons receive information about CO2 levels via relays from the carotid body through the NTS (nucleus of the solitary tract), and directly from chemosensory neurons including the RTN (retrotrapezoid nucleus)^21,41,45,48^, and medullary (Med R) and dorsal (DR) raphe nuclei^43,55,62^. The projections of the PB^FoxP2^ neurons to the preBötzinger complex (PBötZ), rostral ventrolateral medulla (RVLM) and the caudal ventrolateral medulla (CVLM) may mediate the increase in ventilation by increasing respiratory rate (RR)^25,53,64^ and tidal volume (V_T_) or directly by the projections from the Kölliker-Fuse FoxP2 neurons to the phrenic motor^35,53,65^. Mechanical sensory feedback by thoracic stretch receptors during increased ventilatory effort may activate PBel^CGRP^ neurons to cause cortical arousal, via activation of neurons in the basal forebrain (BF), central nucleus of amygdala (CeA), and lateral hypothalamic area (LHA)^19,56^. Selective activation of PB^FoxP2^ neurons may be effective for augmenting breathing in apneas, if combined with ways to suppress the EEG arousal.

Our results of PB^FoxP2^ photo-stimulation that enhanced the breathing frequency but had little effect on the V_T_ are similar to those shown earlier^38–40^ for photostimulation of the PreBotzinger (PBZ) region, where low power stimulation (20HZ at 2 mW) of inhibitory neurons (GABA-ergic and glycinergic) increased the RR but not the V_T_. Similarly, photo-activation of the adjacent catecholaminergic group in the rostral part of the ventrolateral medulla which projects to the respiratory rhythm-generating neurons located in the pre-Botzinger complex^41–45^ also increased the RR in a dose dependent manner^43^. These findings suggest that the PB^FoxP2^ neurons may target the pre-Botzinger complex to cause the increase in RR (Fig. 7).

By contrast, photo-inhibition of the PB^FoxP2^ neurons during a 30 sec exposure to CO2, did not prevent the rise in RR but did diminish the rise in V_T_ and MV, not only during the CO2 exposure but even after the cortical arousal, when the largest increase in V_T_ is generally observed. On the surface it may appear that the increase mainly in RR with photostimulation of the PB^FoxP2^ neurons and decrease mainly in the V_T_ with photoinhibition of the same cells is inconsistent. However, the photostimulation was done while the animals were breathing room air, so the increase in RR cause by the PB^FoxP2^ neurons was not being masked by the contributions of other brainstem sites (such as the retro-trapezoid nucleus, para-facial complex, medullary raphe and the nucleus of solitary tract) that are also engaged by high CO2 levels^41,43, 45–47^. In addition, although the increase in V_T_ was not statistically significant during these brief periods of stimulation, V_T_ changes with hypercarbia lag considerably behind the increase in RR. On the other hand, during 10% CO2 stimulation, when these other CO2-responsive sites were already increasing RR to its maximum,^44, 48–50^ the inhibition of PB^FoxP2^ neurons revealed although they are not needed to reach maximal RR, they are necessary to achieve the maximal increase in ventilatory volume. In other words, brief activation of the PB^FoxP2^ neurons is sufficient to drive RR (but not V_T_) when breathing room air, and necessary to drive increased V_T_ during exposure to high CO2 levels^51–54^. A schematic model describing the proposed neuronal circuit for hypercapnia induced increase in ventilation implicating the role of PB^FoxP2^ neurons is shown in Fig. 7.

In conclusion, our experiments reveal that PB^FoxP2^ neurons likely contribute both to the increases in RR and V_T_ during exposure to CO2 in NREM sleep, but that they are not the only pathway driving these responses (Fig. 7). On the other hand, if ways could be found to augment the PB^FoxP2^ response to CO2, this may be sufficient to avoid hypoventilation and minimize EEG arousals in patients with obstructive sleep apnea. However, activating the PB^FoxP2^ neurons to increase RR by 50% or more above baseline may by itself contribute to EEG arousal. It is not known whether this arousal is due to the mechanical sensory feedback of increased ventilatory effort, or might represent collaterals from the PB^FoxP2^ neurons that indirectly activate PBel^CGRP^ neurons or some other arousal pathways. However, for activation of PB^FoxP2^ neurons to be effective in treating patients with obstructive sleep apnea, it will be important to find ways to suppress the EEG arousal while augmenting the respiratory response to CO2.

## Methods

### Animals:

We employed *Foxp2*^*tm1.1(cre)Rpa*^/J transgenic mice in which IRES-Myc tag-nuclear localization signal (NLS)-cre-GFP-frt-neomycin-frt were introduced just after the termination codon of the mouse Foxp2 gene via homologous recombination. The mutation was created via homologous recombination in (129S6/SvEvTac × C57BL/6) F1-derived G4 embryonic stem (ES) cells. The frt-flanked neomycin cassette was excised through crosses with animals that broadly expressing Flp recombinase. The GFP is believed to be nonfunctional. Resultant mice were backcrossed to C57BL/6J for 9 generations by the donating laboratory to the Jackson laboratory (Strain #:030541; RRID:IMSR_JAX:030541). All transgenic mice used here were heterozygous for the transgene and backcrossed to the C57BL6 strain and wildtype littermates were used as controls. We bred these mice in our animal facility and confirmed their genotype by using a Red Extract N-amp Tissue PCR kit (Sigma Aldrich; Catalog # XNAT-1000RXN) and Cre forward and reverse primers to detect the Cre recombinase gene. Their wildtype litter mates were used as controls, in each experiment.

### Validation of mice:

To test if the Cre expression was eutopic with FoxP2 expression FoxP2-Cre mice from Jackson Labs, we also validated by crossing them to the L10 reporter mice. The cre-positive neurons were labeled with green fluorescent protein (GFP), and when the tissue was immuno-stained for FoxP2, we could observe nearly all green (GFP) neurons expressed labeling for Fox-P2 (red) in their nuclei (supplementary Fig.2a-d). This confirmed that we could reliably use these mice for expressing cre-dependent virus vectors in the FoxP2 neurons in the PB, and then use fiber-photometery or optogenetics to record their activity profiles or manipulate them (examples of cre dependent transfections- Fig.2b, 4b and 6c). PB^Foxp2^ neurons did not overlap with the PB^CGRP^ neurons in the lateral PB.

All mice used in these experiments were male because female mice of the same age are smaller, and including animals of various sizes would introduce noise into analysis of respiratory volumes (which scale with body size) across groups. Animals were maintained on a 12 h light/dark cycle with ad libitum access to water and food and were singly housed after surgery, with ambient temperature of 21–23° C and humidity levels between 40–60%. Male littermates were randomly assigned to the experimental groups. All animal procedures met National Institutes of Health standards, as described in the Guide for the Care and Use of Laboratory Animals, and all protocols were approved by the Beth Israel Deaconess Medical Center Institutional Animal Care and Use Committee.

### Vectors:

For fiber-photometry we used AAV-pSyn-GCaMP6s from Addgene_100843. For optogenetic experiments, we used the excitatory opsin (AAV-EF1a-DIO-hChR2(H134R)-mCherry- AAV-serotype8) and the optogenetic neural silencer AAV-CAG-FLEX-ArchT-GFP (AAV-serotype-8). These viral vectors was procured from the University of North Carolina (UNC) vector core and has been previously used for excitation as well as for silencing neurons and their terminals by us^19,55^. The viral vectors expressing excitatory and the inhibitory opsins were packaged at the UNC vector core.

### Surgery:

Under surgical anesthesia, mice were instrumented for sleep with implantation of EEG and EMG electrodes in addition to implanting either the fiber photometry cannula (unilateral), or integrated grin lens with baseplate (unilateral) or bilateral optical fibers targeted to the PB area (AP: −5.1 to −5.3mm; DV-2.6mm; ML: ±1.3mm). All implants were done after 5 weeks post injection of the injections of the viral vectors in the PB, to ensure optimal expression of the gene expression. After recovery from surgery, mice were recorded for sleep and respiration after acclimatizing them to the recording apparatus for at least once in a week before the actual recordings were performed. We recorded the arousal and respiratory responses to CO2 by placing them in the plethysmograph and recording them for both sleep and breathing by the procedure described previously^19,21,55^.

#### Experiment 1a:

C57BL/6J mice (n=8) were moved from the animal housing room and placed in the plethysmographs where they were exposed continuously to either hypercapnic (21% O_2_; 10% CO_2_; 69% N_2_) or room air (21% O_2_; 0% CO_2_; 79% N_2_) for two hours, after first habituating them to the chamber at room air for two hours. Air or hypercapnic exposures were performed between 10:00 and 12:00. At the end, mice were deeply anesthetized with chloral hydrate (500mg/kg, ip) and transcardially perfused with saline, followed by 10% formalin. Brains were removed, post-fixed overnight immersed in 20% sucrose and cut into four alternate series of 30 micron frozen sections. After immune-staining the tissue for cFos and FoxP2, using specific antibodies as per the methods described below, the cells that were double labeled for cFos and Foxp2 were counted in the PBcl (0.5mmX 0.6mm placed dorsal to the single labeled cFos in the PBel, as in Fig. 1a2–d3) and KF (0.5mm X 0.5 mm placed ventral to the ventral spino-cerebellar tract, as in Fig. 1a1–d1) sub-nuclei, after scanning the slides using a slide scanner (Olympus VS200 slide scanner) and acquiring the images in Olympus OlyVia 3.3 software using square grids. To avoid counting bias, the cell counts were performed by investigators blind to the treatment groups (NL and JDL). The original figures in Fig1a-d were taken by a confocal laser microscope (LasX Leica DMi8 confocal). We counted the sections that were separated by at least 120μm, and this also involved counting only the nuclei for both the FoxP2 and the Cfos, that reduced the chances of counting cells twice. Cell counts were also corrected for the cell size using the Abercrombie correction factor.

#### Experiment 1b:

##### Fiber-photometry implants:

*In vivo* calcium monitoring during sleep-wake and with CO2 exposures was performed using fiber photometry. After 5 weeks of the viral injection of AAV-pSyn-GCaMP6s in the PB, laser light was passed into the brain via low-fluorescence fiber optic patch cord (0.48NA, Doric Lenses) connected to the implanted fiber optic cannula with a metal sleeve (Doric Lenses, MFC_400/430–0.48_5mm_MF-1.25). Using the patch cord, we simultaneously delivered light via LED drivers at 465 nm and 405 nm (Doric Lenses, CA), to measure the calcium-dependent and calcium-independent (UV, isobestic), excitation of the GCaMP. Excitation emission from the GCaMP protein passed back through the fiber optic patch cord and through the fluorescence Mini Cube (Doric Lenses) and detected by a photo-receiver (Doric Lenses). Signals detected by the photo-receiver were transmitted to Axon Digidata 1322A analog-to-digital converter and the signals were acquired using Axoscope software-v10 (Molecular Devices, Foster City, CA, USA), alongside the EEM/EMG and breathing signals. We used exported files to Spike2 and analyzed the respiratory signals using the spike respiratory scripts (Resp80t, Spike2, CED, UK) that were then correlated with the GCaMP activity.

The GCaMP6 raw data was normalized to the baseline fluorescence of each trial to obtain the ΔF/F (change in fluorescence intensity relative to the baseline fluorescence intensity) for different animals. The GCaMP ΔF/F values per second was calculated for 15 s before and during CO2 for each trial and statistically compared, along with RR and VT values for similar periods. The peak values of GCaMP ΔF/F, and the latency to peak during the entire 15s with CO2 exposure were also calculated. Two-way ANOVA was performed to compare the effects between pre and post CO2 exposures on the GCaMP ΔF/F and also for RR and VT.

#### Experiment 1c:

##### GRIN lens implants:

*Foxp2*^*tm1.1(cre)Rpa*^/J mice (heterozygous FoxP2-Cre), after 3weeks of the virus injection (AAV-pSyn-GCaMP6s) in the PB were implanted with a microendoscopic ProView^™^ Integrated Lens 0.6mm × 7.3mm (Inscopix Catalogue #1050–004413) that allowed for visualizing the activity during the lens implant. The lens was targeted to be ~200–300 μm above the neurons using the following coordinates- −5.0mm posterior to bregma, −1.4mm lateral from midline and −2.8 to 3.0 mm ventral to the dura mater. The baseplate provide the interface for attaching the miniature microscope during the calcium-imaging experiments, but during other times a baseplate cover (Inscopix catalogue # 100–000241) was attached to prevent damage to the micro endoscopic lens. Out of approximately 8 mice injected with GCaMP6s virus, 4 had successful implants and were used for the study.

##### Calcium imaging:

We imaged the calcium activity at 5 frames per second, 200-ms exposure time at 20–30% LED power using the miniature microscope from Inscopix (nVista). These parameters caused minimal bleaching, and allowed long term recordings in mice. Mice were recorded for 5 min every hour for correlating the calcium activity to the 12h sleep-wake behavioral data. For correlating to the CO2 induced respiratory changes, mice were recorded in a plethysmograph, where they were subjected to repeated stimulus of 30s of 8% CO2 every 5 minutes, and the imaging was done for 4 trials (~20 min) every hour for 3h after 3–4h of habituation of mice to the recording chamber with every recording. This protocol caused minimum bleaching and allowed repetitive 2–3 recordings in an animal with a week of separation between subsequent recordings.

#### Experiment 2:

Optogenetic activation of the PB^FoxP2^ neurons: For selective activation of the PB^FoxP2^ neurons, we injected an adeno-associated viral vector expressing the excitatory opsin AAV-FLEX-ChR2-mCherry in the PB (AP: −5.1 to −5.3mm; DV-2.6mm; ML: ±1.3mm) and implanted these mice (n=10) with bilateral optical fibers targeting the lateral PB. We also injected some wild type mice (n=3) with AAV-FLEX-mCherry as well, which served as control, we did not observe any expression of ChR2. We recorded these mice for sleep and respiration when they were exposed to the room air as per the method previously described. Mice were subjected to 5 or 10s of 467nm laser stimulus with pulse width of 10ms and with frequencies of either 5Hz or 10Hz or 20Hz, in a random order and with each treatment separated by at least 7–10 days.

#### Experiment 3:

Optogenetic inhibition of the PB^FoxP2^ neurons: A separate set of FoxP2 mice were injected in the PB with AAV-FLEX-ArchT-GFP (n=12), and to test whether PB^FoxP2^ neurons mediate CO2 induced changes in respiration, these mice were bilaterally implanted with optical fibers targeting the PB (AP: −5.1 to −5.3mm; DV- 2.6mm; ML: ±1.3mm) for the inhibition of the PB^FoxP2^ neurons. At 5 weeks post injection, these mice were recorded for sleep and breathing in plethysmography chamber, where the arousal and respiratory responses were assessed while they were subjected to repetitive CO2 stimulations as shown earlier^19,20,55,56^.

### Histology:

At the conclusion of the experiments, the animals were perfused with 0.9% saline followed by 10% buffered formalin while under deep anesthesia. Brains were harvested for analysis of the effective location of the injection site. Brains were kept in 20% sucrose for 2 days and sections were cut at 30mm using a freezing microtome in four 1:4 series.

Immunohistochemistry: C-Fos: Oncogene Sciences, cat # Ab5, rabbit polyclonal, raised against amino acids 4–17 of human c-Fos. This antibody stained a single band of 55 kDa on Western blots from rat brain (manufacturer’s technical information). Sections were processed for detection of c-Fos alone or c- Fos in combination with FoxP2. All incubations were performed on free-floating tissue sections at room temperature. Sections were first incubated overnight in c-Fos antibody. When c-Fos staining was to be combined with FoxP2, we used Rabbit anti-Fos antibody (diluted 1:10K in PBS with 0.2% Triton X-100). After rinsing, sections were incubated in Alexa488 (green fluorescence) conjugated donkey anti Rabbit-IgG (Invitrogen, A11055) at 1:500 in PBS containing 0.2% Triton-X and 2.5% normal donkey serum for three hours. After rinsing in PBS sections were next incubated overnight with rabbit anti-FoxP2 (diluted 1:5000 in PBS with 0.2% Triton X-100 and 2.5% normal donkey serum). After rinsing the next day sections were incubated in Cy3 (red fluorochrome) conjugated donkey anti-rabbit IgG (Jackson Immuno-Research Labs, code#111-165-003) or with streptavidin-pacific blue (1:200, ThermoFischer, cat-S11222) after reacting the tissue to the appropriate biotinylated secondary antibody.

Mice injected with either GCamP6s or ArchT or ChR2, were immunostained either for GFP (Rabbit anti-GFP, 1:10K, Molecular Probes Cat# A-11122, RRID:AB_221569) or with mcherry (rabbit anti DsRed. 1:2K, Clontech, Cat-632496) as per standard immunohistochemistry protocols described previously^19,55^. These were then double stained for FoxP2 using either anti-rabbit (Rabbit anti-Foxp2, 1:10K, Abcam Cat# ab16046, RRID:AB_2107107) or anti-sheep antibodies (Sheep anti FoxP2, 1:5K, R and D Systems Cat# AF5647, RRID:AB_2107133). We used rabbit polyclonal antibody raised against a Synthetic peptide conjugated to KLH derived from within residues 700 to the C-terminus of Human FOXP2 (AbCam) and for the sheep polyclonal antiserum raised against recombinant human FoxP2 isoform 1, Ala640-Glu715, Accession # O15409 (R&D Systems) and these have been previously used and validated by others^57–59^. Neither of these antibodies showed immunostaining when the primary antibodies were omitted, and when the tissue from control mice was used that were not injected with viral vector. Some of the brains (n=3) from WT mice were injected with CTb and were immunostained using Goat anti CTb (1:30K, Cat# 703, RRID: AB_10013220, List Biological Laboratories Inc., CA). Sections for double staining for GFP, mcherry, CTb or FoxP2 were incubated in fluorescent-labeled secondary antibodies (Alexa- 488 at 1:200 or Alexa- Cy3 at 1:200; Catalog #- A32790 and A10521, RRID- AB_2762833 and RRID- AB_2534030, Molecular probes, Thermo-Fischer Scientific) or with streptavidin-pacific blue (1:200, ThermoFischer, cat-S11222) for 2h and cover-slipped with fluorescence mounting medium (Dako, North America). When acquiring confocal images, sometimes pseudocolors were used to enhance clarity.

### Fluorescent In situ hybridization (FISH using RNA Scope):

We identified FoxP2 neurons by using *Foxp2*^*tm1.1(cre)Rpa*^/J mice crossed with R26-lox-STOPlox-L10-GFP reporter mice (FoxP2-L10, n=3), and labeled for *Calca* (the gene for CGRP) by using a set of FISH probes with RNAScope in brain sections from the KF and PBcl areas. The brain was sectioned at 30 μm and sections were mounted on glass slides in RNAs-free conditions, and RNA scope was performed using the multiplex fluorescent reagent Kit V2 (Cat# 323100, Advanced Cell Diagnostics, Hayward, CA). Brain sections on the slides were pretreated with hydrogen peroxide for 20 min at room temperature and then with target retrieval reagent for 5 minutes (at temperature above 99°C), followed by dehydration in 90% alcohol and then air-dried for 5 minutes. This is followed by a treatment with protease reagent (Protease III) for 30 minutes at 40°C. After rinsing in sterile water, sections were hybridized in CGRP/Calca FISH probe (smFISH probe: Mm- Mm- Calca-tv2tv3-C1- Mus musculus, Calcitonin-related polypeptide alpha, transcript variant 2 mRNA; probe region: 63 – 995 Accession No. NM_001033954.3; Catalog# 420361; Advanced Cell Diagnostics, Hayward, CA) for 2 hours at 40°C, and this probe has been used previously to selectively label CGRP on the brain tissue^60,61^. Sections were then incubated in 3 amplification reagents (AMP) at 40°C (AMP1 for 30minutes, AMP2 for 30 minutes and AMP3 for 15 minutes) followed by Horse radish peroxidase – C1amplification at 40°C for 15 minutes. Sections were then incubated in tyramide signal amplification (TSA) reagents with Cy3 fluorophore (Cat# NEL744001KT, Perkin Elmer, 1:1000) for 30 min to amplify and visualize CGRP mRNA in red. In the final step, sections were subjected to HRP blocking for 15 min at 40°C. After each step, sections were washed with 1X wash buffer provided in the kit. Following the CGRP RNAscope in-situ hybridization, immuno-labeling of GFP was performed on the same sections, as in-situ procedure quench the green fluorescence. For this, the brain sections were incubated in rabbit anti-GFP (1:1500), (Cat#A6455; Lot#1220284; Molecular probes) for overnight at 4°C, washed in PBS (3X2 minutes) and then incubated in secondary antibody (Alexa Fluor- 488 Donkey anti Rabbit, Life Technologies, Cat# A-21206) for 2h at room temperature. Finally, the slides were dried and cover-slipped with Dako fluorescence mounting medium (Cat# S302380–2, Agilent, CA), and scanned for analysis.

## Data acquisition

All recordings were done at five weeks after injection of the viral vectors. All sleep and respiration recordings were done in a plethysmography chamber (unrestrained whole-body plethysmograph, Buxco Research Systems) which allowed us to record the breathing of the mouse while continuously monitoring the gas in the chamber. Electroencephalogram (EEG) and electromyogram (EMG) were recorded using Pinnacle preamp cables connected to an analog adaptor (8242, Pinnacle Technology). Gas levels in the chamber were continuously monitored using CO2 and O2 monitors from CWE, Inc (Ardmore, PA, USA). EEG, EMG, respiration, and CO2 and O2 levels were fed into an Axon Digidata 1322A analog-to-digital converter and the signals were acquired using Axoscope software- v10 (Molecular Devices, Foster City, CA, USA), or by acquired by the 1401 (CED, Cambridge, UK) and Spike2 ver.7 (CED, Cambridge, UK). Mice were connected to cables for sleep recording as well as with the fiber-optic cables connected to the pre-implanted glass fiber in mice, for transmitting the laser light.

Mice were also placed in the plethysmography chamber beginning at 9:00 A.M. for 6 h during their lights-ON and behaviorally inactive period, on each test day for these recordings. Here, they underwent either the Laser-ON or Laser-OFF protocols, separated by a week and in random order.

During the Laser-ON protocol, either 473nm or 593nm laser was ON for 5, 10 0r 60s followed by 5 mins off. With 473nm, the photo-stimulations were done at either 5, 10 or 20Hz with pulse width of 10ms, and these stimulations were done for either 5 or 10s on different days, with a period of 6–7days between each treatment. During photo-activation using the 473nm laser, only normocapnia air was used in the chamber. The inhibitory 593nm laser stimulations were continuous for 60s, and preceded each 30s of CO2 stimulus by 20s and lasted 10s after the hypercapnia stimulus. In the Laser-OFF condition, everything was the same, except that the laser light was not turned on. The gas input for the plethysmograph was switched either to normocapnic air (21% O2, 79% N2) or hypercapnic air (10% CO2, 21% O2, and 69% N2) for 30 sec with 5 minutes in between the two hypercapnic stimuli. For both the photo-activation and inhibition experiments, trials were analyzed for latency to arousal and respiratory changes only for those epochs where the mouse was in NREM sleep for at least 30 s before the stimulus onset.

Laser light: Mice were allowed at least 2d to acclimate to fiberoptic cables (1.5 m long, 200 μm diameter; Doric Lenses, Quebec, QC, Canada) and connecting interfaces coated with opaque heat-shrink tubing before the experimental sessions. During Laser-ON experiments, light pulses were programmed using a waveform generator (Agilent Technologies, catalog #33220A, CA, USA) to drive either 10ms of 473nm (blue laser, Laser Glow, Toronto, ON, Canada) pulses at 5, 10 or 20Hz, or drive the orange-yellow light laser (593 nm; Laser Glow, Toronto, ON, Canada) to be continuously on for 60s beginning 20 s before the onset of the CO2 stimulus. We also used the splitter - TM105FS1B (Thorlabs, NJ) to split the laser stimulus for bilateral activation or inhibition. We adjusted the laser such that the light power exiting the bilateral fiber-optic cables was 8–10 mW, and this was checked before and after the experiment. The light power estimated at the PB is less than10 mW/mm^2^ (www.stanford.edu/group/dlab/cgi-bin/graph/chart.php), and a similar range has been used by most researchers and by us earlier^19,55,62,63^. Note that this is probably a high estimate because some light is probably lost at the interface between the fiber-optic cable and the implanted optic-fiber.

## Data analysis

### Latency and respiratory data analysis:

EEG arousals in response to CO2 were identified by EEG transition from NREM to a waking state, which was usually accompanied by EMG activation, as described previously. The latency of all the EEG arousals after onset of stimulation were scored and were compared across the Laser-ON and Laser-OFF days. Respiratory data was analyzed by running the respiratory script in the Spike2 (CED, UK) software, which performs breath by breath analysis for many respiratory parameters such as RR, V_T_ and MV. For both latency and respiratory data, the ranges for analysis were selected by individuals that were blind to the treatment groups, who based the selection off the following criteria: 1. Trials with at least 30 seconds of NREM sleep before CO2/stimulation; 2. Select 5 breaths before CO2 or laser stimulation, during CO2/stimulation before arousal and then at post- arousal for respiratory analysis; 3. Exclude trials with REM sleep.

### Fiber photometry data:

The voltage GCaMP and UV signals were Gaussian low-pass filtered at 4 Hz, and saved for offiine analysis. Both signal channels (465 and 405 nm) were monitored continuously throughout recordings, with the 405 nm signal (UV) used as an isobestic control. Signals detected with 405 nm wavelength light are not calcium-dependent and are indicative of background fluorescence or motion artifacts. A change in fluorescence (ΔF/F) was calculated by normalizing F to baseline fluorescence. For generating heat maps, min-max normalization was performed that causes linear transformation and the data is scaled in the range (0,1).

### Inscopix calcium-image processing:

Calcium recording files were spatially filtered and motion corrected to correct the rigid brain movements using the Inscopix data processing (IDPS ver1.6). To extract the calcium activity traces from the individual cells, we used manually drawn small regions of interest. Raw traces were converted to ΔF/F (F-F_baseline average_/F_baseline average_), where F was the fluorescent at any given point and F_baseline average_ was the average baseline fluorescence. These baseline image calculations were performed by the IDPS to derive ΔF/F value for each cell.

### Statistical analysis:

All statistical analyses were performed using SigmaPlot 12.3 (Systat Software, Inc.). For statistical comparisons, we first confirmed if the data meets with the assumptions of the ANOVA, then either one way or two-way ANOVA was performed to compare the effects between various treatment groups. If differences in the mean values among the treatment groups were greater than would be expected by chance; then all pairwise multiple comparisons were performed using the Holm-Sidak method. The F and P values are described in the results section with details of the statistical tests also given in the respective figure legends and represented in the figures. The ‘n’ is reported in the figures and results and represents the number of animals, and the error bars represent mean± SEM. Using SigmaPlot 12.3, we also tested the sample size and power of the tests post hoc and found that the power of each statistical test was at least 80% at alpha= 0.05, suggesting adequate sample sizes for all the experiments. A probability of error of less than 0.05 was considered significant.

## Data Availability

All data generated to support the findings of this study are available from the corresponding author upon reasonable request.
